# Decoupling species richness variation and spatial turnover in beta diversity across a fragmented landscape

**DOI:** 10.7717/peerj.6714

**Published:** 2019-04-10

**Authors:** Guang Hu, Maxwell C. Wilson, Jianguo Wu, Jingjing Yu, Mingjian Yu

**Affiliations:** 1 School of Civil Engineering and Architecture, Zhejiang Sci-Tech University, Hangzhou, China; 2 School of Life Sciences, Arizona State University, Tempe, AZ, USA; 3 School of Sustainability, Arizona State University, Tempe, AZ, USA; 4 College of Life Sciences, Zhejiang University, Hangzhou, China

**Keywords:** Area effect, Environmental filtering, Isolation, Land-bridge islands, Plant functional type, Thousand Island Lake

## Abstract

**Background:**

How habitat fragmentation affects the relationship between local richness and the variation in community composition across space is important to both ecology and conservation biology, but this effect remains poorly understood.

**Methods:**

Here, we present an empirical study to address this topic in a fragmented landscape, the Thousand Island Lake (TIL), an artificial land-bridge island system with more than 1,000 islands, which provides an “experimental” fragmented landscape with a homogeneous matrix and similar successional history. We measured species composition and plant functional type (PFT) on 29 islands, and tested the effects of island area and isolation on the relationship between α- and β-diversity. General Linear Models were applied to test the impact of habitat fragmentation. In addition, variation partitioning was used to decouple α-diversity dependent and α-diversity independent spatial turnover in β-diversity of the plant community and across different PFTs.

**Results:**

We found habitat fragmentation influences β-diversity of plants primarily by modifying local α-diversity, not spatial turnover in the TIL system. We also found area-dependent environmental filtering and differential plant responses across functional types were the most likely underlying driving mechanisms.

**Discussion:**

These results highlight the importance of hierarchical linkages between components of biodiversity across scales in fragmented landscapes, and have practical conservation implications.

## Introduction

Habitat loss is one of the most serious threats to biodiversity on a global scale (Pimm & Raven, 2000). One of the real-world consequences of habitat loss often is the breaking up of large, contiguous patches into smaller, more numerous, more isolated patches (Collinge, 2009). Studies of these processes, often and controversially given the single moniker of “habitat fragmentation,” have been fraught with definitional, conceptual, and methodological challenges (Collinge, 2009; Fahrig, 2003), limiting our ability to mechanistically disentangle the impacts of habitat fragmentation on biological communities across scales (Didham, Kapos & Ewers, 2012). For example, recent work has made great strides in increasing our understanding of how increased area, isolation, and edge effects will impact the biological communities in fragments (Haddad et al., 2015) and how the spatial component of habitat fragmentation alters regional biodiversity patterns (Fahrig, 2017). However, much of this work is focused on a single spatial scale, and we know relatively little about how communities that are on increasingly smaller patches in increasingly isolated landscapes will differentiate themselves from one another as fragmentation-mediated processes interact across spatial, temporal, and organizational scales (Wilson et al., 2016).

In considering this question, two divergent possibilities become apparent at the patch-scale. One possibility is the habitat fragmentation mediates species-specific filters across gradients of patch size and isolation, resulting in deterministically derived nested communities wherein community structure is richness dependent (Dennis, Hardy & Dapporto, 2012; Patterson & Atmar, 1986). This process is also called “environmental filtering,” which removes species less adapted to abiotic restrictions (Keddy, 1992), such as space and nutrient reductions, enhanced edge effects, and drought in the fragmented landscape (Hu et al., 2011). Another possibility is that individuals will be either equally impacted by fragmentation’s effects, as in neutral theory (Chave, 2004; Hubble, 2001), or that species will sort themselves based on local selective pressures that are largely independent of fragmentation at the patch-scale (e.g., “species sorting”) (Holyoak et al., 2005), either of which will result in stochastic assemblies at the patch-scale once the species-area relationship is accounted for. This dichotomized view of deterministic vs. stochastic patch-level community controls, while perhaps overly simplistic (Holyoak et al., 2005), provides a useful mental model begetting testable predictions for assessing the importance of fragmentation-mediated selective pressures in metacommunity dynamics.

However, our ability to assess either of these hypotheses has been constrained by the fact that the common metrics used to estimate community similarity across patches (e.g., Sørenson’s or Jaccard’s dissimilarity metrics) are intrinsically richness dependent (Baselga, 2010; Si, Baselga & Ding, 2015; Wu et al., 2017). While recent methodological advancements have greatly eased this problem by partitioning the variation in community structure between sites into richness-dependent and independent components (Legendre, 2014), the relative efficacy of these methods remains controversial (Baselga & Leprieur, 2015; Legendre, 2014). By partitioning the richness-dependent and independent components in beta diversity, Bishop et al. (2015) reported that the species turnover and nestedness of montane ant communities resulted in opposite trends in the variation of community composition with increasing elevation. A meta-analysis found species turnover dominated beta diversity of a variety taxa in island systems in 94% of global datasets (Wu et al., 2017).

In our initial work in the Thousand Island Lake (TIL) region, we found island area directly affected species richness while isolation indirectly affected species composition (Yu et al., 2012). Furthermore, patterns of nestedness generated species-specific responses to different island attributes (Hu et al., 2011) and higher beta diversity was found on smaller islands due to environmental heterogeneity rather than ecological drift (Liu et al., 2018b). However, these studies did not consider the respective linkages between the ecological mechanisms, island attributes, and richness-(in)dependent components in beta diversity. To close this gap, we empirically disentangled the relative importance of richness-independent spatial turnover and richness variation in influencing the variation in community composition across patches in a contemporary fragmented landscape (a land-bridge island system). We also wanted to test three hypotheses in the study: (1) the relationship between α- and β-diversity would vary across islands with different attributes; (2) differences in environmental filtering across scales would be the main mechanism shaping the α- and β-diversity relation; (3) different responses of plant functional types (PFTs) to fragmentation would also play a key role in the relationship between α- and β-diversity. Using both richness-dependent and richness-independent measures of community similarity, along with a suite of biogeographical and ecological attributes, we then explored the underlying mechanisms driving the variation in community composition, elucidating the relative importance of richness vs. turn-over in a real world fragmented landscape.

## Materials and Methods

### Study site

The study was conducted in a land-bridge island system in a man-made reservoir, the TIL in East China. The climate is subtropical monsoon with an average annual temperature of 17 °C and precipitation of 1,430 mm (Editorial committee of development of Xin’an River, Xin’an River Development Corporation, 2009). The inundation following the Xin’an River Dam construction for power generation in 1959, resulted in 1,078 fragmented islands (>0.25 ha) when the water reached its highest level (108 m a.s.l.). The areas of most islands are less than one ha. Currently, most islands are covered with forests (∼90%) dominated by Masson pine (*Pinus massoniana*) in the canopy and broad-leaved plants in the sub-canopy and understory. Forests were formed by secondary succession through regeneration after dam construction (Wilson et al., 2016). The majority of TIL has been protected by government and the vegetation has experienced few human disturbances over the past several decades. The TIL system may be considered as an “experimental” fragmented landscape with homogeneous matrix and synchronous successional history (Hu et al., 2011; Wilson et al., 2016; Yu et al., 2012).

### Data collection

#### Field work for plant communities

**Figure 1:**
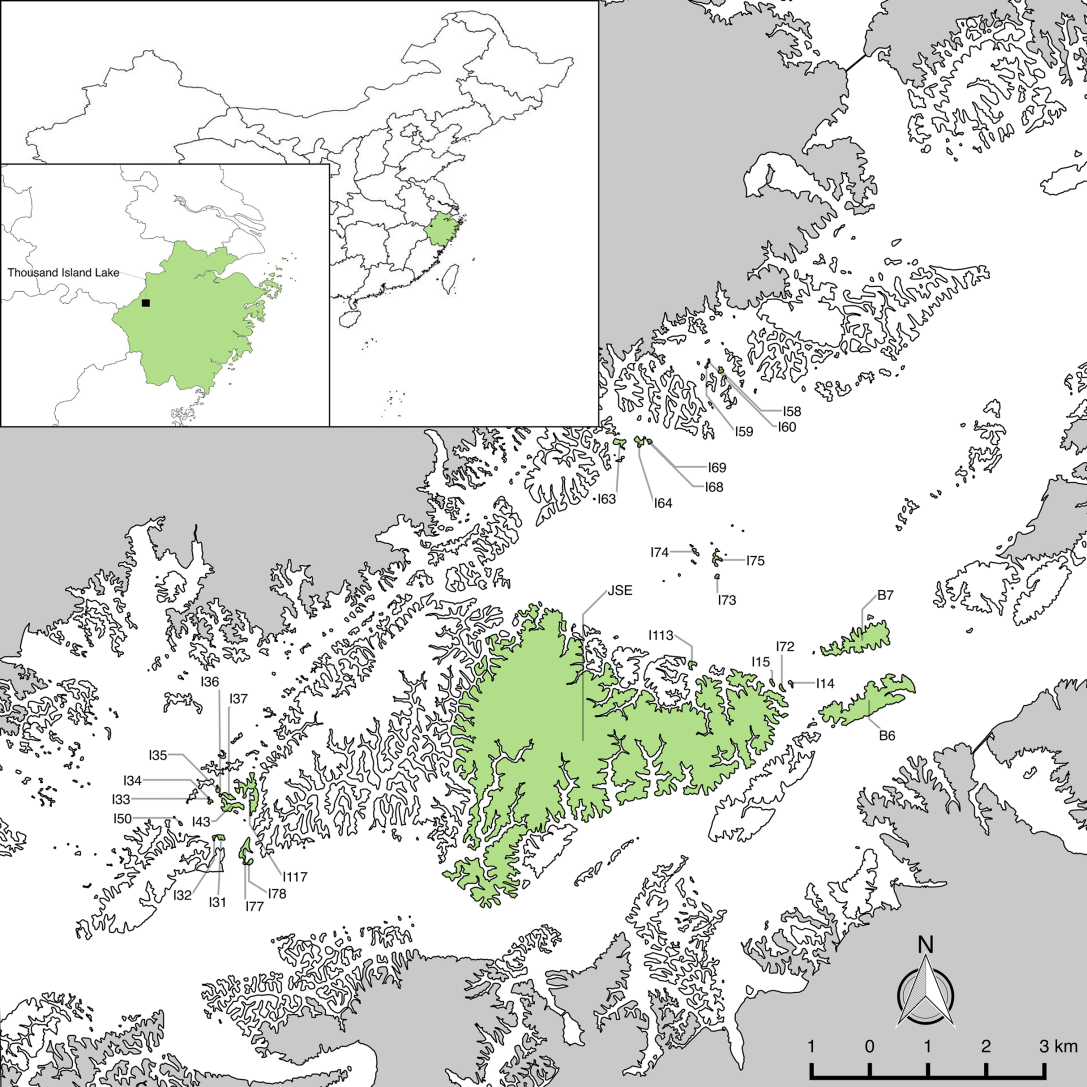
The 29 study islands in the Thousand Island Lake, East China. Island and mainland boundaries were digitalized using SPOT-6 satellite imagery in ArcMap v10.3 (http://desktop.arcgis.com/en/arcmap/). Final map production was completed in QGIS v2.8 (www.qgis.org). Maps of China and Zhejiang Province are based on the Natural Earth 1:10,000,000 cultural and physical data sets, which are part of the public domain and available at http://www.naturalearthdata.com/.

We established long-term forest plots on 29 islands (Fig. 1) with little human disturbance across different gradients of area and isolation in 2009–2010 (Hu, Feeley & Yu, 2016). To assess whether the 29 studied islands are representative of the larger fragmented landscape, Wilson (2018) compared the distributions of island area, distance to mainland (DM) and distance to nearest neighbor island of the 29 studied islands and 510 islands in the fragmented landscape using the Kolmogorov–Smirnov test. Results showed there are no significant differences between the distributions of the 29 islands and all islands in the landscape for island area (*D* = 0.20, *P* = 0.22). Plot establishment and data collection followed the protocol for plot censuses established by the Center for Tropical Forest Science—Forest Global Earth Observatory network (Condit, 1998; Condit et al., 1999). The census of plots took one of three shapes: (1) cover the entire island (island area ≤1 ha); (2) 0.5 ha sampling plot (island area one to five ha) and (3) one ha plot (island area >5 ha) on these study islands. All woody individuals with diameter at breast height (DBH) >1 cm were measured in height and DBH , and were identified to species. Rarefaction analyses indicated that sampling protocols were sufficient to capture most woody plant species on the islands (Yu et al., 2012).

In total, 186,781 individuals of 76 woody plant species were recorded on the 29 islands.

#### Plant functional types

To test different responses of species to fragmentation, we classified the plant species into PFTs, with PFTs containing the species with similar resource usage strategies and disturbance tolerance. Different PFTs have showed different performances in the species-area relationship (Hu et al., 2012) and community succession (Liu et al., 2018a) in TIL. Eight plant attributes, maximal height, leaf area, special leaf area (SLA), leaf thickness, leaf dry material content, chlorophyll content, twig dry material content, and wood density, which are directly linked to plant defense strategies (Cornelissen et al., 2003; Pérez-Harguindeguy et al., 2013), were measured for each species according to the general protocols described with Cornelissen et al. (2003) and Pérez-Harguindeguy et al. (2013). The maximal height of each species was recorded from the highest individual in the plots. Leaf traits were measured with 10 fully expanded current-year leaves in the sun-exposed conditions from five mature trees distributed in our study system. Stem traits were measured using 10 branches from five mature trees after oven drying to constant mass at 80 °C and divided by fresh volume determined by water displacement.

We classified all woody plant species recorded on 29 islands into PFTs by a clustering analysis based k-means partitioning (Caliński & Harabasz, 1974) using the eight plant traits. The effectiveness of this classification was tested using Detrended correspondence analysis (Appendix S1; Fig. S1). To facilitate the analysis of data and interpretation of results, we classified these species into four plant functional types (Appendix S1; Table S1). Compared with our previous studies about the responses of plant species to fragmentation, PFT1 (evergreen) and PFT2 (deciduous) are common species that are less sensitive to habitat fragmentation, whereas PFT3 and PFT4 are rare species that are more sensitive to habitat fragmentation (Hu et al., 2012).

#### Island and landscape attributes

Wilson (2018) identified the landscape attributes of the study islands from high resolution remote sensing imagery (SPOT-6). Three attributes, island area, distance to nearest island (DNI) and DM were calculated using a combination of FRAGSTATS (v4.2) and ArcGIS (v10.4). These two distances can be contributed to different isolation effects. DNI indicated the spatial assemblage of study islands with others, and DM indicated the direct connectivity between target island and species pool. There was no significant Pearson’s correlation between these two distances.

To test the area and isolation effects on diversity, we classified the study islands according to three different sets of criteria: island area, distance to the nearest neighboring island, and DM. This produced the following three pairs of classes: (1) large islands (area >1 ha) and small islands (area ≤1 ha); (2) isolated islands (DNI >50 m) and clumped islands (DNI ≤50 m); (3) far islands (DM >2,000 m) and near islands (DM ≤2,000 m). According to the criteria, we compare and contrast the 29 islands in three ways: 11 large islands vs. 18 small islands; 10 isolated islands vs. 19 clumped islands; and 19 far islands vs. 10 near islands. Spearman’s rank correlation analysis showed these categories are independent.

### Data analysis

#### α- and β-diversity

Species richness was used to represent α-diversity of all plants and PFTs on each island.

To calculate the variation in community composition for each island pair (e.g., β-diversity) within these island groups, we used two indices. First, we calculated classical Jaccard’s dissimilarity index (β_J_) to represent β-diversity composed of both richness variation and spatial turnover. Second, we calculated Raup-Crick metric (β_RC_) as described with Chase et al. (2011) to represent the richness-independent component of β-diversity (e.g., species replacement). The advantages of β_RC_ are twofold. First β_RC_ statistically disentangles variation in spatial turnover between samples from variation in α-diversity. Second, β_RC_ separates deterministic and stochastic processes on β-diversity (Segre et al., 2014) based on a null-model approach (Chase et al., 2011), while controlling for species loss caused by habitat fragmentation. The value of β_RC_ ranges from −1 to 1. Values approaching −1 indicate higher similarity between two communities than expected, suggesting that either deterministic competition or environmental filters shared across samples dominates the system at the scale of analysis. Values approaching 1 indicate that the two communities have higher dissimilarity than expected, suggesting that deterministic environmental filters not shared across samples favor divergent species composition or that there is low dispersal among fragments (dispersal limitation). Values close to 0 indicate that assemblies are highly stochastic and that dispersal is frequent across fragments.

The null model applied in β_RC_ computation was determined for each island by randomly sampling species from species pool (all species recorded in our field) under two constraints: (1) the simulated species richness of each island was fixed and equaled the observed richness; and (2) the probability of each species to be sampled was proportional to its observed abundance (the r1 model described in Wright et al. (1997)). We repeated this simulation 1,000 times to calculate β_RC_.

#### Statistical analysis

We fitted generalized linear models (GLM) with a Gaussian link function to predict the β-diversity of all plants and four PFTs based on the three island attributes. A backward stepwise regression was then used to identify the best-fit models with variable selection and model evaluation were both based on Akaike information criterion values. The contribution of each island attribute to predicting species diversity was determined by calculating the proportion of the deviance explained, similar to *R*^2^. We then used variation partitioning base on redundancy analysis ordination (Borcard, Legendre & Drapeau, 1992) to estimate the independent contributions of species richness variation and β_RC_ (spatial turnover) to the variation in β_J_ for the whole system, the islands groups, and each PFT.

This analysis was applied to the whole plant community and each PFT. All statistical analyses were conducted with R 3.3.2 (R Develpment Core Team, 2015).

## Results

### Pattern of α-diversity

Species richness variation between island pairs was significantly influenced by island area for all plants and individual PFTs (Table 1). More species were recorded in islands in the large island group. DNI and DM slightly affected richness variation of different PFTs.

**Table 1:** Results from generalized linear models (GLMs) and stepwise regression of island attributes for predicting the plant diversity in the Thousand Island Lake.

Species diversity	Island attributes^†^	Estimate	SE	*P*-value	Deviation explained (%)
Richness variation					
All plants	Area	7.62 × 10^−2^	6.83 × 10^−3^	<0.001	42.15
PFT1	Area	6.42 × 10^−2^	1.22 × 10^−2^	<0.001	14.28
	Area × DM	−1.70 × 10^−5^	8.33 × 10^−6^	0.043	0.54
PFT2	Area	7.74 × 10^−2^	7.28 × 10^−3^	<0.001	40.00
PFT3	Area	8.42 × 10^−2^	4.69 × 10^−3^	<0.001	47.58
	DM	−4.02 × 10^−5^	1.25 × 10^−5^	0.001	1.60
PFT4	Area	3.23 × 10^−2^	1.45 × 10^−2^	0.027	13.65
	Area × DNI	1.24 × 10^−3^	3.80 × 10^−4^	0.001	0.33
	Area × DNI × DM	−5.39 × 10^−7^	2.06 × 10^−7^	0.009	1.62
Spatial turnover (β_RC_)					
All plants	Area	1.73 × 10^−2^	5.60 × 10^−3^	0.002	2.83
	DM	3.85 × 10^−5^	1.45 × 10^−5^	0.008	1.66
PFT1	Area	4.63 × 10^−2^	1.44 × 10^−2^	0.001	4.68
PFT2	Area	4.41 × 10^−2^	6.05 × 10^−3^	<0.001	10.75
	DM	4.72 × 10^−5^	1.57 × 10^−5^	0.003	1.97
PFT3	Area	2.08 × 10^−2^	5.22 × 10^−5^	<0.001	3.85
PFT4	Area	6.30 × 10^−2^	1.32 × 10^−2^	<0.001	15.12
	DM	1.16 × 10^−4^	3.33 × 10^−5^	0.001	0.035
	Area × DNI	5.42 × 10^−4^	2.26 × 10^−4^	0.017	0.45
	Area × DM	−1.87 × 10^−5^	8.46 × 10^−6^	0.033	0.63
	DNI × DM	−1.94 × 10^−6^	5.22 × 10^−7^	<0.001	3.24
β-diversity (β_J_)					
All plants	Area	7.53 × 10^−2^	9.98 × 10^−3^	<0.001	16.09
	DM	7.22 × 10^−5^	2.04 × 10^−5^	<0.001	0.67
	Area × DM	−2.19 × 10^−5^	6.86 × 10^−6^	0.002	2.05
PFT1	Area	6.74 × 10^−2^	1.08 × 10^−2^	<0.001	10.54
	DM	8.07 × 10^−5^	2.21 × 10^−5^	<0.001	1.06
	Area × DM	−2.17 × 10^−5^	7.44 × 10^−6^	0.004	1.82
PFT2	Area	6.76 × 10^−2^	1.08 × 10^−2^	<0.001	15.91
	DM	5.13 × 10^−5^	2.18 × 10^−5^	0.019	0.08
	Area × DM	−2.02 × 10^−5^	7.40 × 10^−6^	0.006	1.54
PFT3	Area	2.26 × 10^−2^	5.37 × 10^−3^	<0.001	5.69
	DM	3.78 × 10^−5^	1.43 × 10^−5^	0.009	1.98
PFT4	Area	2.31 × 10^−2^	6.46 × 10^−3^	<0.001	3.35

**Notes:**

Only attributes retained in the best-fit models are shown. Area was log-transformed. Variable selection and model estimation were based on the Akaike information criterion (AIC).

† DNI, Distance to nearest neighbor island.

DM, Distance to Mainland.

### Pattern of β-diversity

**Figure 2:**
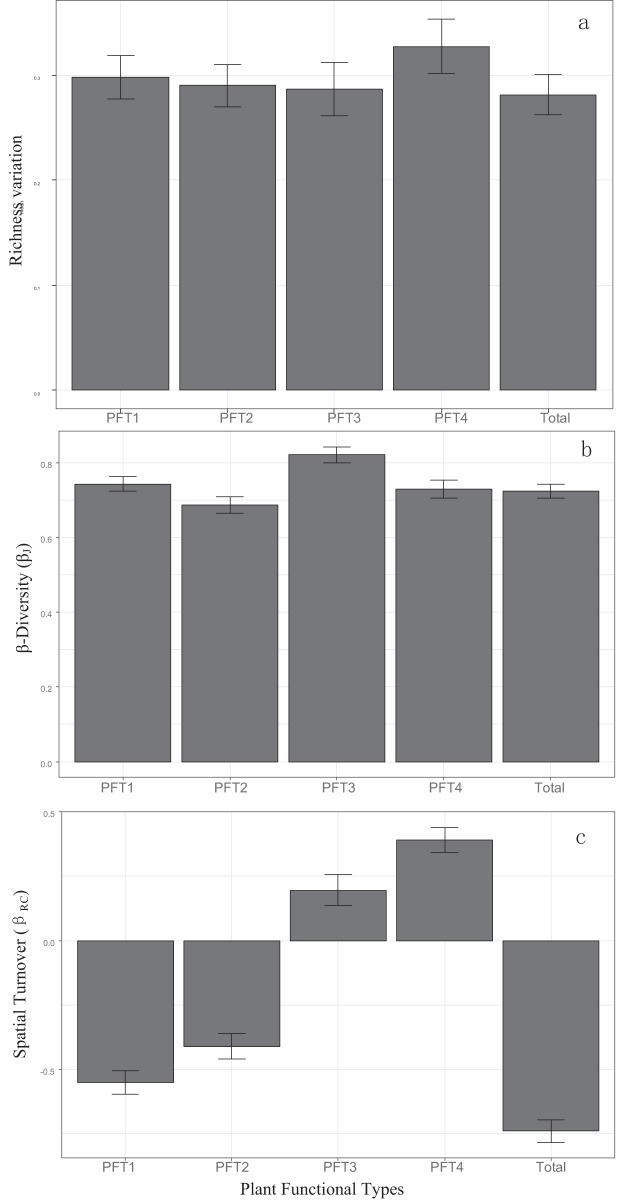
Patterns of (A) α-diversity, (B) β-diversity and (C) spatial turnover in all plant and four PFTs. Bars represented 95% confidence levels.

β_RC_ value of all plants, PFT1 and PFT2 were negative (Fig. 2C), and had significant correlation with island areas (Table 1). PFT3 and PFT4 had positive β_RC_ values. β_RC_ of PFT3 only correlated with area, while β_RC_ of PFT4 was affected by area, DM and the area-isolation interaction.

β_J_ of all plants, PFT1, PFT2, and PFT3 increased with the increasing island area and DM. But the area-DM interaction had negative effects (Table 1). DNI had no significant effects on β_J_.

### Contributions of richness variation and spatial turnover to β-diversity

**Figure 3:**
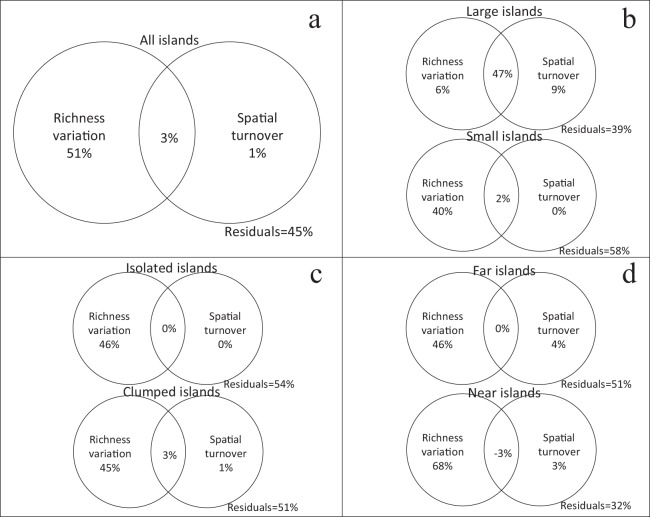
Relative contribution of richness variation and spatial turnover in β-diversity of four island categories ((A) all islands; (B) large vs. small; (C) isolated vs. clumped; (D) far vs. near) based on variation partitioning. The independent and combined explanatory powers are shown in parentheses.

Using variation partitioning, richness variation independently explained 51% of the β-diversity (β_J_) for all islands, while spatial turnover only explained 1% (Fig. 3A). In the small island group, richness variation was also the main factor explaining the β-diversity. But in the large island group, the independent explanation of spatial turnover increased to 9%, while richness variation only explained 6% (Fig. 3B). Isolation has no effect on the relative contribution of richness variation and spatial turnover in β-diversity (Fig. 3C and 3D).

**Figure 4:**
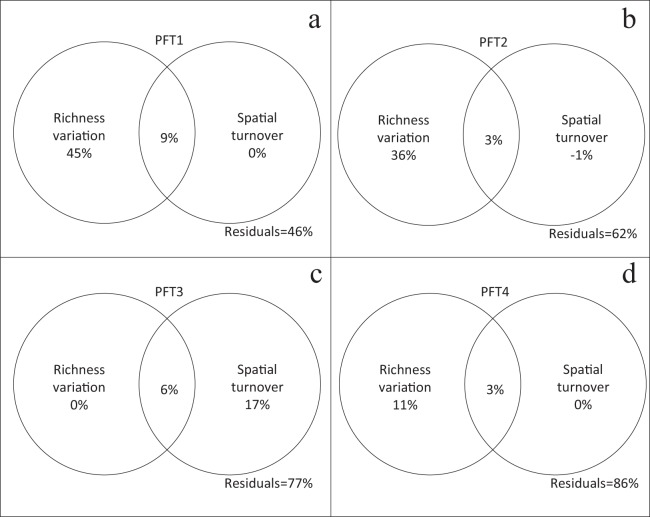
Relative contribution of richness variation and spatial turnover in β-diversity of (A) PFT1, (B) PFT2, (C) PFT3 and (D) PFT4 based on variation partitioning. The independent and combined explanatory powers are shown in parentheses.

Plant functional types had different performances in the variation partitioning analysis. β-diversity (β_J_) of PFT1, PFT2, and PFT4 were only explained by richness variation (Figs. 4A, 4B and 4D). But spatial turnover explained 17% variation in β-diversity for PFT3, regardless richness variation (Fig. 4C).

## Discussion

Our study was able to distinguish between the relative contributions of variation in richness (e.g., α-diversity) and spatial turnover to the variation in community structure between patches (e.g., β-diversity) in a fragmented landscape, providing new insights into how these three variables are related and how their relationship may be influenced by patch and landscape attributes.

### Effects of island area on α- and β-diversity

In the TIL decreased patch size due to habitat loss has a strong negative impact on species richness, whereas increased isolation has no significant impact on species richness (Wang et al., 2010; Yu et al., 2012). In our previous studies, we found the decline of habitat heterogeneity caused by area loss was the main driver of this decreased α-diversity for multiple taxa, such as plants, birds, and lizards (Wang et al., 2010; Yu et al., 2012).

Island area also influenced the variation in community structure, involving the α-independent spatial turnover (e.g., β_RC_) and α-dependent dissimilarity (e.g., β_J_) across islands. Variation partitioning showed that the variation in species richness explained nearly 50% of the variation in β-diversity (Fig 3A). The variance explained by each factor in GLM analysis also supports this finding, with island area explaining more of the deviation in GLMs predicting β-diversity than other parameters (Table 1). Communities on the island pairs with greater area variation were more dissimilar, suggesting an area effect working on β-diversity across islands. This relationship was also found in small mammals in Atlantic fragmented forests, which was explained by the higher vulnerability of these species and increasing environmental heterogeneity across small fragments (Pardini et al., 2005). We speculate area effect on plant β-diversity was caused by habitat divergence and following environmental filtering (Myers et al., 2015) on the different islands. This speculation is informed from our previous studies, which showed environmental filtering was the main driver of community assembly in seedling-sapling transition (Hu, Feeley & Yu, 2016).

However, we also found evidence that environmental filtering drove the species assembly at different scales. The large island group had relatively high α-diversity and high variation explained by spatial turnover. We also found that GLM coefficients associated with area were positive when predicting β_RC_ (e.g., as the difference is area between island pairs becomes larger the island communities become more dissimilar independent of richness variation). Together this suggests that higher habitat heterogeneity within large islands could be driving community assembly on these islands. In contrast, habitat diversity is lower within, but higher across the small islands (Liu et al., 2018b). As mentioned above, our GLMs showed that the difference is island area is positively related to community dissimilarity at the island scale once richness variation was eliminated. Combined with relatively low α-diversity and low variation explained by spatial turnover this suggests that environmental filters shared across small islands play an important role in driving community structure. Our previous studies have also found steeper species-area relationship in the more fragmented landscapes with smaller islands (Hu et al., 2012), which illustrated the severe richness variation occurred in small island groups, and drove the β-diversity pattern.

### Effects of island isolation on α- and β-diversity

Isolation has no or slight effects on species richness in this system, as demonstrated by several studies in TIL (Hu et al., 2011; Hu, Feeley & Yu, 2016; Yu et al., 2012). However, isolation, especially the DM, significantly affected the β-diversity including β_J_ and β_RC_, albeit with relatively low amounts of deviance explained. That species similarity and spatial turnover were both affected by DM illustrates that most species on islands were strongly linked with the species pool on the surrounding mainland. Selective immigration of pollinators or seed dispersers could be one explanation for this β-diversity pattern (Wang, Chen & Ding, 2011). However, different from oceanic islands, islands far from the mainland in TIL are located in the center of the lake. It caused the difference of habitat quality between near islands (close to lakeshore) and far islands (distant to lakeshore). The worse habitat quality on near islands was influenced by the disturbance from surrounding lakeshore. Higher density of beetles founded in the far islands (Zhou et al., 2017) also support this explanation. In TIL region, we speculated that habitat quality is more important than immigration, which caused DM positively affect the β-diversity.

### Responses of PFTs

There was little difference in richness variation and β-diversity between PFTs. The only significant differences in the richness-independent spatial turnover were between common species (PFT1, 2) and rare species (PFT3, 4), illustrating different community assembly processes for each PFT. Lower spatial turnover of common species and high spatial turnover of rare species (Fig. 2C) suggests that deterministic competition exclusion or shared environmental filters across fragments drove the assembly process (Chase et al., 2011) of common species, while specific habitat demands or low dispersal among fragments (dispersal limitation) drove the assembly of rare species.

Generalized linear models analysis illustrated that PFT4 was impacted by the largest number of fragmentation altered parameters. This suggests that plants with high SLA leaves and low tolerance to disturbance could suffer severe impacts from habitat fragmentation. Positive β_RC_ values of PFT3, suggests that the specific habitat requirements regulated the occurrence of these species. PFT3 also showed a unique relationship between α-diversity and β-diversity relative to other PFTs, with spatial turnover of PFT3 primarily driving β-diversity (Fig. 4C). Specific habitat requirements and dispersal limitation might enhance the spatial turnover of PFT3 among islands. That greater habitat dissimilarity was found to cause higher β-diversity among smaller islands in TIL (Liu et al., 2018b) also supports this point. Similar to the performance of the whole communities, negative β_RC_ values of suggests that spatial turnover of PFT1 and PFT2 was determined by the shared environmental filtering (Chase et al., 2011) across islands. Thus PFTs differ significantly in which drivers of β-diversity control their response to habitat fragmentation in this landscape.

Our finding that β-diversity for plant communities in the TIL system is α-dependent could be viewed as support for previous work that has shown communities in the TIL to be nested (Hu et al., 2011; Wang et al., 2010). However, similar decompositions of β-diversity in the TIL system for bird, lizard, and spider communities have shown that species-turnover is the dominant component of β-diversity for these taxa (Si, Baselga & Ding, 2015; Wu et al., 2017). Future work should aim to understand why the variation in community structure varies so widely across taxonomic groups in this system.

## Conclusions

The effects of habitat fragmentation on biodiversity represent one of the most important topics in landscape ecology and conservation biology. However, little is known about how α- and β-diversity are related in fragmented landscapes and how patch attributes may alter this relationship. To fill this gap, our study offers the following important findings:

1. Variation in α-diversity influences β-diversity more strongly than spatial turnover in plant communities across this fragmented landscape. Island area primarily influences β-diversity by modifying local α-diversity, not spatial turnover.
2. Environmental filtering drives species assembly at different scales along an area gradient. Higher spatial turnover within large islands is due to higher habitat heterogeneity. In contrast, in the small island groups, habitat heterogeneity is rather limited and environmental filtering decreases spatial turnover, thus richness variation becoming the main driver of β-diversity.
3. Common species suffering less environmental filtering are widely dispersed, and decrease the contribution of spatial turnover in β-diversity. However, rare species are more sensitive to environmental filtering, and consequently lead to the higher contribution of spatial turnover in β-diversity.

Our study highlights the importance of the relationship between α- and β-diversity in fragmented landscapes. Our results have broad implications for biological conservation, emphasizing the complex, scale-dependent nature of the relationship between local and regional diversity, and require us to rethink how habitat fragmentation alters biodiversity across scales. Our results suggest that traditional α-dependent dissimilarity indices may overestimate the diversity across sites in some cases because relatively little spatial turnover (β_RC_) means that most species on small islands are the same. In other words, species dissimilarity (β_J_) is mostly attributed to species richness variation. Thus, conserving most large fragments and some small fragments with highest local richness should be the main aim of natural conservationist and local government in the TIL region. Special attention should be paid to protecting both special species composition and habitat heterogeneity on larger islands. Future studies are needed to test the generality of these findings in fragmented landscapes and for different taxa across multiple scales and organizational levels.

## Supplemental Information

10.7717/peerj.6714/supp-1 Supplemental Information 1 Appendix S1: Classification and description of four plant functional types.

## Additional Information and Declarations

### Field Study Permissions

The following information was supplied relating to field study approvals (i.e., approving body and any reference numbers): Field experiments were approved by the local manager, the Xin’an River Development Corporation.

### Data Availability

The following information was supplied regarding data availability: The data of plant communities used in this study was drawn from: Hu G, Feeley KJ, Yu M. 2016. Habitat fragmentation drives plant community assembly processes across life stages. *PLOS ONE* 11(7): e0159572. DOI 10.1371/journal.pone.0159572
