# Supplementary material for: Decoupling species richness variation and spatial turnover in beta diversity across a fragmented landscape
Source: PeerJ. 2019 Apr 10;7:e6714. doi: 10.7717/peerj.6714 (PMC6462183; doi:10.7717/peerj.6714)
Supplement: Supplemental Information 1 [file peerj-07-6714-s001.pdf]

PeerJ

SUPPLEMENTARY INFORMATION

Decoupling species richness variation and spatial turnover in beta diversity across a fragmented landscape

Guang Hu, Maxwell C. Wilson, Jianguo Wu, Jingjing Yu, Mingjian Yu

APPENDIX S1: Classification and description of four plant functional types.

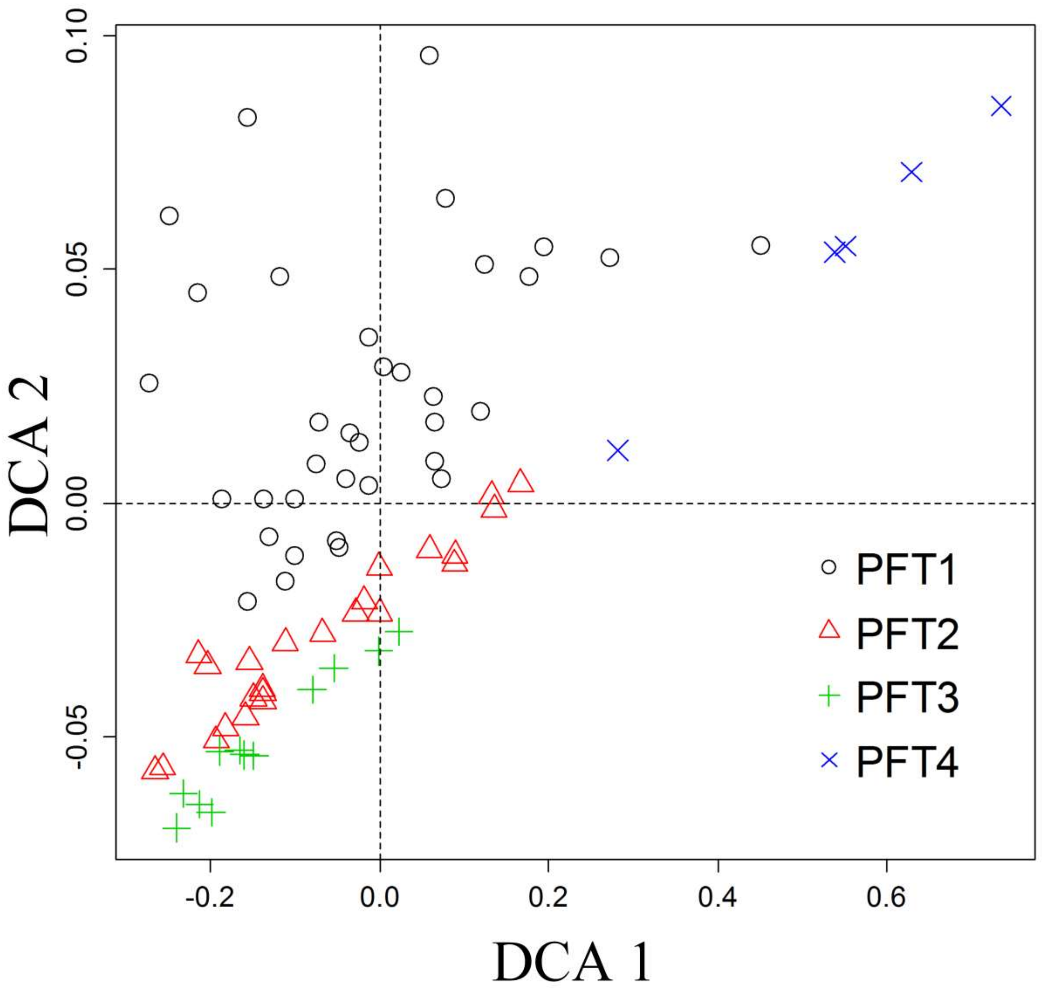

Fig. S1 Detrended Correspondence Analysis (DCA) was used to test the classification of plant functional types.

Table S1 Description of four plant functional types

PFTs	Richness	Description
PFT1	36	Evergreen, low SLA, shade tolerant, common species
PFT2	24	Deciduous, low SLA, shade tolerant or intolerant, common species
PFT3	11	Deciduous, low SLA, shade intolerant, rare species
PFT4	5	Deciduous, high SLA, shade intolerant, rare species
